# Inclusion in the World Health Organization Model List of Essential Medicines of Non-Vitamin K Anticoagulants for Treatment of Non-Valvular Atrial Fibrillation: A Step Towards Reducing the Burden of Cardiovascular Morbidity and Mortality

**DOI:** 10.5334/gh.608

**Published:** 2020-08-06

**Authors:** Ezequiel J. Zaidel, Xinyi Leng, Abiodun Moshood Adeoye, Ferdous Hakim, Biraj Karmacharya, Asim Katbeh, Lis Neubeck, Stephanie Partridge, Pablo Perel, Mark D. Huffman, Mariachiara Di Cesare

**Affiliations:** 1 Cardiology Department, Sanatorio Güemes, and Pharmacology Department, School of Medicine, University of Buenos Aires, AR; 2 Department of Medicine and Therapeutics, The Chinese University of Hong Kong, Shatin, Hong Kong SAR, CN; 3 Cardiovascular Unit, Department of Medicine, University College Hospital, Ibadan/College of Medicine, University of Ibadan, Ibadan, NG; 4 Cardiovascular Genetics and Genomic Research Unit, Institute of Cardiovascular Diseases, Faculty of Clinical Sciences, College of Medicine, University of Ibadan, NG; 5 Research and Publication Unit, World Health Organization, BD; 6 Department of Public Health, Public Health and Community Programs, Kathmandu University School of Medical Sciences, NP; 7 International PhD Programme in Cardiovascular Pathophysiology and Therapeutics, CardioPaTh, BE; 8 School of Health and Social Care, Edinburgh Napier University, GB; 9 The University of Sydney Susan Wakil School for Nursing and Midwifery, Charles Perkins Centre, Sydney, AU; 10 Westmead Applied Research Centre, Faculty of Medicine and Health, The University of Sydney, AU; 11 Department of Non-communicable Disease Epidemiology, London School of Hygiene and Tropical Medicine, GB; 12 Northwestern University Feinberg School of Medicine, Departments of Preventive Medicine and Medicine and Center for Global Cardiovascular Health, Chicago, Illinois, US; 13 The George Institute for Global Health, University of New South Wales, Sydney, AU; 14 Department of Natural Science, School of Science and Technology, Middlesex University London, UK

**Keywords:** non-valvular atrial fibrillation, non-vitamin K antagonist oral anticoagulants (NOACs), Essential Medicine List, stroke, prevention

## Abstract

Non-vitamin K antagonist oral anticoagulants (NOACs) represent a paradigm shift in the treatment of non-valvular atrial fibrillation (AF) with major practice guidelines around the world recommending NOACs over vitamin K antagonist oral anticoagulants for initial treatment of AF for stroke prevention. Here we describe the evidence collated and the process followed for the successful inclusion of NOACs into the 21st WHO Model List of Essential Medicines (EML).

Individual NOACs have been reported to be non-inferior or superior to warfarin in preventing stroke and systemic embolism in eligible AF patients with a reduction in the risk of stroke and systemic embolism and a lower risk of major bleeding in patients with non-valvular AF compared with warfarin in both RCTs and real-world data.

The successful inclusion of NOACs in the WHO EML is an important step forward in the global fight against cardiovascular morbidity and mortality, especially in low- and middle-income countries, where the burden of disease is high and limited access to diagnosis and treatment translates into a higher burden of morbidity, mortality, and economic costs.

## Introduction

Non-vitamin K antagonist oral anticoagulants (NOACs) represent a paradigm shift in the treatment of non-valvular atrial fibrillation (AF) for stroke prevention since, as opposed to vitamin K antagonist oral anticoagulants (VKAs), they do not require routine International Normalized Ratio (INR) testing and have far fewer drug-drug and drug-food interactions [1]. NOACs have shown a favourable balance between efficacy and safety compared with VKAs in treating eligible AF patients, with significant reductions in stroke, intracranial haemorrhage, and mortality [2]. As a result, major clinical practice guidelines around the world recommend NOACs over warfarin for initial treatment of AF for stroke prevention [1,3,4]. As costs have substantially declined since the introduction of NOACs in 2009, these drugs represent an effective, cost-effective alternative to VKAs for patients with AF, especially for those with limited access to health care for whom routine INR monitoring is difficult. A 2018 modelling analysis forecasted future trends of ischaemic stroke and death rates in AF patients by comparing the scenario of unchanged oral anticoagulant use in East Asia (45% of patients on VKAs; 55% on NOACs) with the alternative scenario of a continued increase in NOAC use. NOACs uptake to 90% would help prevent an estimated 206,315 ischaemic strokes and 139,353 deaths from 2031 to 2050 in East Asia compared with unchanged use of oral anticoagulants in this region over the same period [5]. Similarly in Europe, the introduction of NOACs in 2010 has been estimated to have led to >88,000 fewer strokes, thromboembolisms and deaths each year. As an example, a model assuming that the rate of edoxaban use were to increase from 11% in 2013 to 75% in 2030 with the remaining NOAC-eligible population taking warfarin, an additional 12,000 cases of stroke, thromboembolism and death would be avoided annually [5,6]. Information regarding increased OAC use or NOAC uptake in low- and middle-income countries or low resource settings is scarce, but currently VKA therapy has limitations due to cultural and geographical barriers. As an example, among anticoagulated patients, those living in low income countries have the worst time in therapeutic range [7]. Therefore, NOACs have been increasingly used in these situations. For instance, the proportion of NOACs usage in AF patients has been drastically increasing in China in recent years. In a large registry study analysing 189,006 prescriptions of anticoagulants for AF patients at 67 hospitals in 5 major cities in China, the percentage of patients receiving NOACs increased from below 2% in 2012 to 28% in 2017 [8].

In this paper we describe the evidence collated and the process followed for developing an application for the inclusion of NOACs into the 21st WHO Model List of Essential Medicines (EML); further information is available in the full application [7]. Essential medicines are defined as those medicines ‘that satisfy the priority healthcare needs of the population. Essential medicines are intended to be available within the context of functioning health systems at all times, in adequate amounts, in appropriate dosage forms, with assured quality and adequate information, and at a price the individual and the community can afford’ [9]. The WHO EML is used as a guide for the development of national essential medicine lists and represents a critically important tool for the development of national health system policies and practices. Every two years since 1977 the WHO Expert Committee on Selection and Use of Essential Medicines reviews submission to add, delete, or revise the EML and Model List of Essential Medicines for Children (EMLc) based on the most up-to-date scientific evidence on the efficacy, safety and cost-effectiveness of medicines.

## Application for Inclusion of NOACs into the WHO Model List of Essential Medicines

A first attempt to include NOACs into the WHO EML was undertaken in 2014 [10]. Despite the available evidence from randomized controlled trials (RCTs) in terms of efficacy and safety of NOACs versus warfarin, the application was rejected because the Committee expressed concern that the application’s supporting data came almost exclusively from RCTs, highlighting concerns that trial populations may not be representative of patients who would receive such treatment in real-world practice [11]. Since 2014, there has been a substantial increase in real-world data published with large-scale registry studies, databases of insurance claims, and systematic reviews and meta-analysis of NOACs in special-risk populations (e.g. elderly, renal impairment, and high HAS-BLED score). Additional safety concerns related to the lack of antidotes have also been addressed since the introduction of NOAC-specific antidotes. Increasing data on costs and cost-effectiveness also helped overcome some of the reasons for the rejection of the 2014 application.

The successful application for the inclusion of NOACs (Dabigatran as representative of the pharmacological class) into the 21st WHO EML was led by the World Heart Federation Emerging Leaders GOALPoST (improving Global access to Oral AnticoaguLants to Prevent Stroke in aTrial fibrillation) team, comprised of academics from different fields and different regions in the world who have a goal to increase global access to NOACs among eligible AF patients, especially in resource-limited regions. Together, this team collated relevant evidence regarding NOACs versus VKAs in AF from pivotal RCTs, large-scale real-world registries, and cost-effectiveness studies, which supported a robust application for the inclusion of NOACs in EML, which was approved by WHO in 2019 [7,12]. Moreover, to achieve the goal, the study team members are conducting field work in Nigeria and Nepal to better understand perceptions and experiences of NOAC usage among AF patients and other stakeholders. The Rapid Assessment Protocol for Insulin Access [13] to explore the path of the drugs from their production to their final use amongst patients was used, with the aim to identify bottlenecks that may hinder the access to NOACs among patients. Results of this study will inform key actors of the nature of those barriers, leading to an increase in the use of oral anticoagulants for AF and specifically NOACs, and along with this, many strokes, bleedings, and deaths might be avoided, especially in resource limited settings.

### Efficacy and safety of NOACs versus VKAs: evidence from RCTs and real-world data

**Figure 1:**
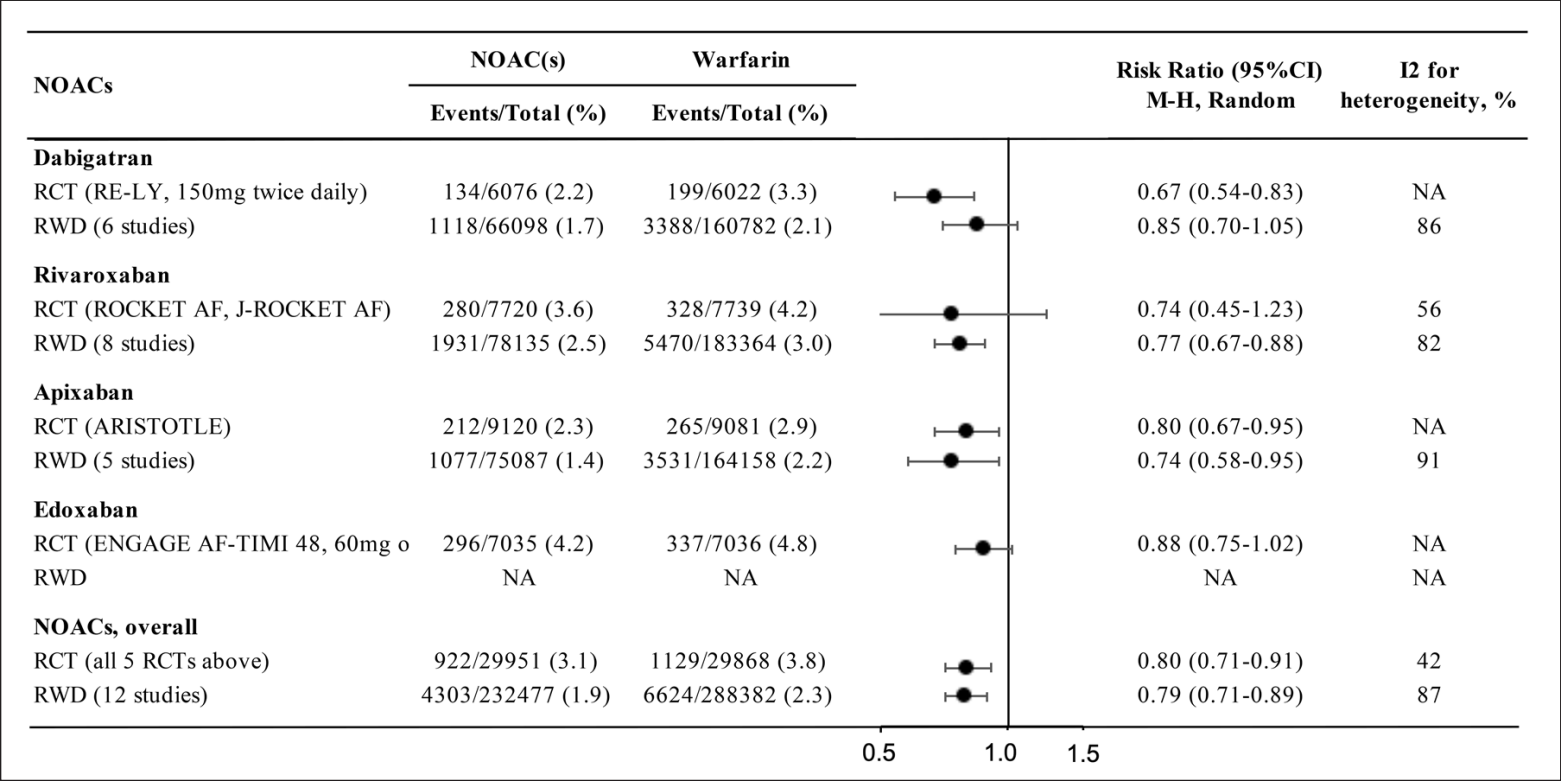
The efficacy of NOACs versus warfarin in preventing stroke and systemic embolism in patients with non-valvular AF based on data from RCTs and studies reporting real-world data (RWD).

**Figure 2:**
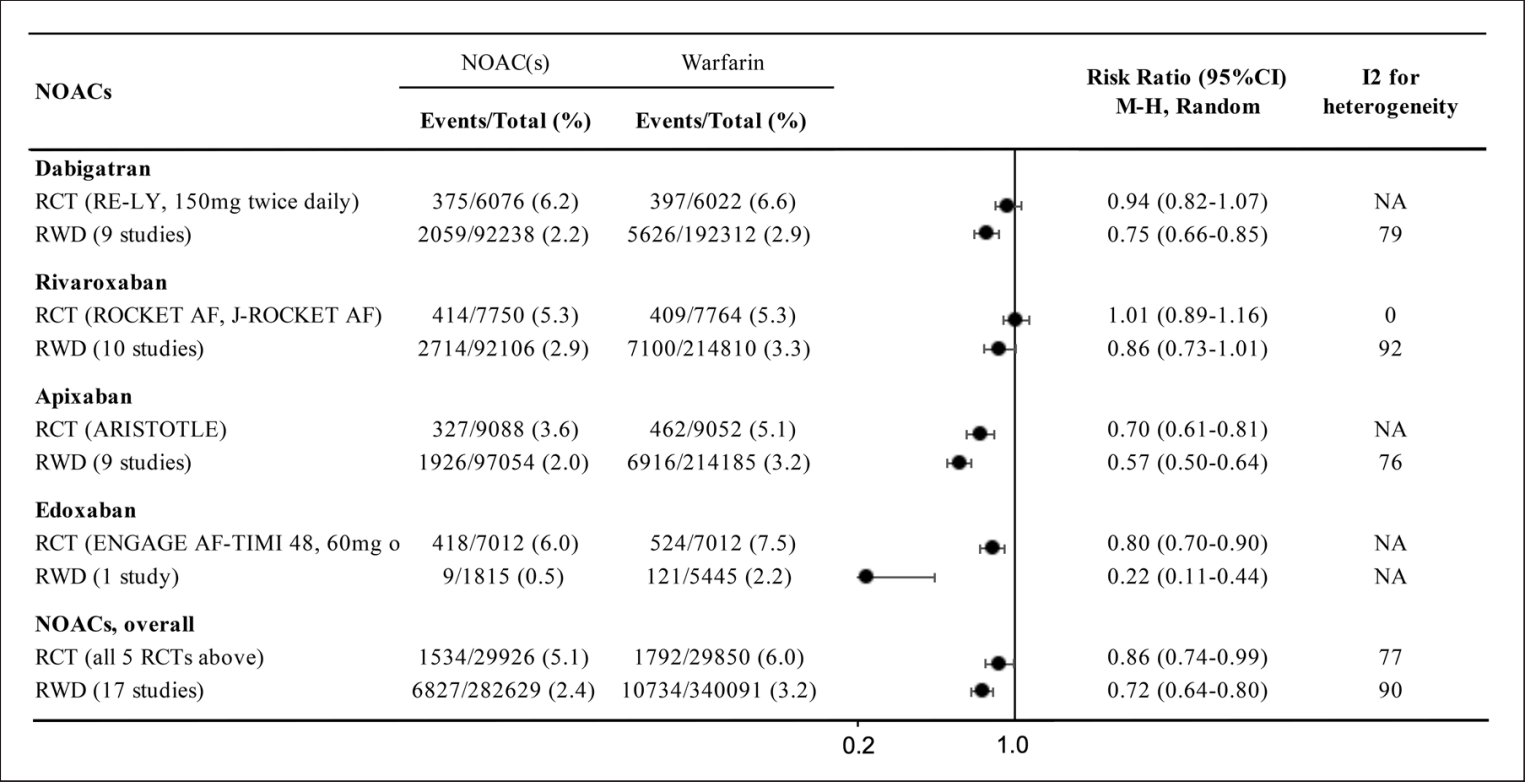
The risks of major bleeding in non-valvular AF patients treated with NOACs versus warfarin based on data from RCTs and studies reporting real-world data (RWD).

A brief summary of the characteristics of the 4 NOACs are provided in Table 1. The efficacy and safety of NOACs (Dabigatran, Apixaban, Rivaroxaban, Edoxaban) versus VKAs (mostly warfarin in RCTs and in clinical practice) in preventing stroke and systemic embolism in non-valvular AF patients have been investigated in individual pivotal RCTs including Randomized Evaluation of Long-Term Anticoagulation Therapy (RE-LY) [14], Rivaroxaban Once Daily Oral Direct Factor Xa Inhibition Compared with Vitamin K Antagonism for Prevention of Stroke and Embolism Trial in Atrial Fibrillation (ROCKET-AF) [15], ROCKET AF in Japanese patients (J-ROCKET AF) [16], Apixaban for Reduction in Stroke and Other Thromboembolic Events in Atrial Fibrillation (ARISTOTLE) [17], and Effective Anticoagulation with Factor Xa Next Generation in Atrial Fibrillation–Thrombolysis in Myocardial Infarction 48 (ENGAGE AF-TIMI 48) [18] trials, in numerous systematic reviews and meta-analysis, as well as in real-word data studies. We updated currently available evidence regarding the efficacy and safety of NOACs, individually or as a group, compared with warfarin in treating non-valvular AF patients, in the application to include NOACs in the WHO EML [7]. The efficacy and safety outcomes of individual and all NOACs versus warfarin from RCTs and from real-world studies are shown in Figures 1 and 2, respectively.

**Table 1:** Brief summary of the characteristics of the 4 NOACs.

NOAC	Mechanism	Pivotal Trial	Year	Type of drug	Oral dosage form	Low or adjusted dose

Dabigatran	IIa inhibitor	RE-LY	2009	Capsule	150 mg bid	110 mg bid
Rivaroxaban	Xa inhibitor	ROCKET	2011	Tablet	20 mg qd	15 mg qd
Apixaban	Xa inhibitor	ARISTOTLE	2011	Tablet	5 mg bid	2.5 mg bid
Edoxaban	Xa inhibitor	ENGAGE	2013	Tablet	60 mg qd	30 mg qd

Individual NOACs have been reported to be non-inferior or superior to warfarin in preventing stroke and systemic embolism in eligible AF patients. In a meta-analysis of the 5 RCTs (59,819 patients), NOACs reduced the risk of stroke and systemic embolism by 20% in patients with non-valvular AF compared with warfarin (RR 0.80, 95% CI 0.71–0.91; p = 0.0003; Figure 1). A similar result in terms of direction and magnitude was observed in meta-analysis of real-world data from 12 studies with 520,859 patients (RR 0.79, 95% CI 0.71–0.89; p < 0.001; Figure 1). No real-world data were available from large-scale studies for the associations between edoxaban versus warfarin and risk of stroke and systemic embolism [7].

The safety of NOACs has been evaluated by the risk of major bleeding. In meta-analysis of the 5 major RCTs (59,776 patients), NOACs were associated with a lower risk of major bleeding compared with warfarin in patients with non-valvular AF (RR 0.86, 95% CI 0.74–0.99; p = 0.04; Figure 2). Further, a meta-analysis of 17 studies with real-world data (622,720 patients) demonstrate a similar direction and possible larger magnitude of effect on major bleeding among non-valvular AF patients treated with NOACs compared with warfarin (RR 0.72, 95% CI 0.64–0.80; p < 0.001; Figure 2), though these results may be partially driven by residual confounding in these non-randomized comparisons.

Unlike RCTs, there is greater risk of bias and between-study heterogeneity in the design and patient characteristics of real-world studies [7]. However, patients involved in observational studies often represent a broader, more diverse, and more complex patient population treated with NOACs compared with highly selected patients in RCTs. Indeed, synthesized findings from these studies have further corroborated conclusions about the safety and efficacy of NOACs versus warfarin in preventing stroke and systemic embolism in patients with non-valvular AF derived from RCTs.

### Evidence for the use of NOACs in elderly patients

A persistent concern about NOACs has been their safety in elderly AF patients, but recent data have been reassuring. First, data from the Prevention of thromboembolic events-European Registry in Atrial Fibrillation (PREFER-AF) registry showed net clinical benefit of anticoagulation versus no anticoagulation, among all age subgroups in elderly (>75 years old) patients [19]. Second, a review in 2016 including data of subgroups of patients over 75 years old in published RCTs, showed safety and efficacy of NOACs over warfarin, except for an increased risk of bleeding with dabigatran at the higher dose (150 mg versus 110 mg bid) [20]. Third, an analysis of US Medicare claims of patients >75 years old demonstrated that patients receiving apixaban experienced lower rates of stroke and bleeding than warfarin [21]. Fourth, an analysis of the oldest old (>90 years) patients with AF from Taiwan demonstrated NOACs as a favorable choice over warfarin for anticoagulation with net clinical benefit [22]. Therefore, current evidence supports NOACs as the best choice for elderly AF patients while avoiding the regimen of dabigatran 150 mg bid.

### Cost-effectiveness of NOACs versus VKAs

As a relatively new group of drugs, the costs of NOACs were expected to be higher than conventional treatment with warfarin. Numerous studies have investigated if the higher direct costs of replacing VKAs with NOACs would be offset by reduced healthcare costs related to reduced stroke, bleeding, and their consequences. A 2016 systematic review concluded that NOACS were cost-effective in several countries, independent of the health systems, direct costs of NOACs and VKAs, and costs of relevant diseases [23]. These authors defined a drug as cost-effective when the incremental cost-effectiveness ratio was below the willingness-to-pay value, ranging from: $US50,000 and $US100,000 in the United States; $CAD30,000 and $CAD50,000 in Canada; £20,000 and £30,000 in the UK; and €20,000 and €79,000 in the remaining European nations. The studies included were on average of high quality. Our study team updated the systematic review in the application to include NOACs in the WHO EML, including 64 cost-effectiveness analyses from 28 high- and middle-income countries, but no analyses have yet been reported from low-income countries. Most studies used the same criteria to define cost-effectiveness, but newer cost-effectiveness analyses from the US have included costs from healthcare resource use and real-world data from health systems to determine the rate of stroke and bleeding rather than using data solely from RCTs, which have strengthened findings from earlier studies. All studies to date have demonstrated that NOACs are cost-effective in treating eligible patients with AF compared to VKAs [7].

By the time of EML submission, NOACs were widely available in high-income countries and increasing elsewhere, with monthly costs ranging from 60 USD in India or Brazil to 300 USD in the US. In some countries, generic formulations of dabigatran are available [7].

### Antidotes for NOACs

The introduction of idarucizumab, a monoclonal antibody against dabigatran that is widely available, and the more recently FDA-approved andexanet alfa, an antidote for apixaban and rivaroxaban, have responded to concerns about the need for an urgent reversal of NOACs, in the rejection of the previous (2014) application to include NOACs in WHO EML. Reversing NOACs with andexanet alfa seems to be associated with better outcomes in intracranial hemorrhages than reversing warfarin with plasma or 4-factor prothrombin complex concentrate [24,25].

## Conclusions

One of the 9 global targets to achieve a 25% reduction in the risk of premature mortality from non-communicable diseases by 2025 (the WHO 25 × 25 goals) focuses on increased coverage of essential medicines for non-communicable diseases, specifically – 80% availability of affordable basic technologies and essential medicines, including generics, required to treat major noncommunicable diseases in both public and private facilities [26]. Successful inclusion of NOACs in the WHO EML is an important step forward in the global fight against cardiovascular morbidity and mortality, primarily through stroke prevention, especially in low- and middle-income countries, where the burden of disease is high and limited access to diagnosis and treatment translates into a higher burden of morbidity, mortality, and economic costs compared with higher-income countries hindering the achievement of the Sustainable Development Goals 3 and 10 [27].

With the inclusion of NOACs in the WHO EML, the next step is to translate this evidence synthesis report and global application to national EML applications for better access and affordability of NOACs. The authors herein advocate national and regional healthcare authorities to implement strategies and policies to enhance availability and accessibility to NOACs for eligible AF patients. Moreover, the identification of obstacles to NOAC access and development of a rapid assessment tool for NOAC treatment in low-income settings can further improve the accessibility to NOACs among those populations and people in greatest need.
